# Interpretation of microbiota-based diagnostics by explaining individual classifier decisions

**DOI:** 10.1186/s12859-017-1843-1

**Published:** 2017-10-04

**Authors:** A. Eck, L. M. Zintgraf, E. F. J. de Groot, T. G. J. de Meij, T. S. Cohen, P. H. M. Savelkoul, M. Welling, A. E. Budding

**Affiliations:** 1 Department of Medical Microbiology and Infection Control, VU University medical center, Amsterdam, The Netherlands; 2 Informatics Institute, University of Amsterdam, Amsterdam, The Netherlands; 3 Department of Gastroenterology and Hepatology, VU University medical center, Amsterdam, The Netherlands; 4 Department of Pediatric Gastroenterology, VU University medical center, Amsterdam, The Netherlands; 5 Canadian Institute for Advanced Research, Toronto, Canada; 6 Department of Medical Microbiology, Maastricht University medical center, Maastricht, The Netherlands

**Keywords:** Microbiota, Inflammatory bowel disease (IBD), Supervised classification, IS-pro, Machine learning

## Abstract

**Background:**

The human microbiota is associated with various disease states and holds a great promise for non-invasive diagnostics. However, microbiota data is challenging for traditional diagnostic approaches: It is high-dimensional, sparse and comprises of high inter-personal variation. State of the art machine learning tools are therefore needed to achieve this goal. While these tools have the ability to learn from complex data and interpret patterns therein that cannot be identified by humans, they often operate as black boxes, offering no insight into their decision-making process. In most cases, it is difficult to represent the learning of a classifier in a comprehensible way, which makes them prone to be mistrusted, or even misused, in a clinical environment. In this study, we aim to elucidate microbiota-based classifier decisions in a biologically meaningful context to allow their interpretation.

**Results:**

We applied a method for explanation of classifier decisions on two microbiota datasets of increasing complexity: gut versus skin microbiota samples, and inflammatory bowel disease versus healthy gut microbiota samples. The algorithm simulates bacterial species as being unknown to a pre-trained classifier, and measures its effect on the outcome. Consequently, each patient is assigned a unique quantitative estimation of which species in their microbiota defined the classification of their sample. The algorithm was able to explain the classifier decisions well, demonstrated by our validation method, and the explanations were biologically consistent with recent microbiota findings.

**Conclusions:**

Application of a method for explaining individual classifier decisions for complex microbiota analysis proved feasible and opens perspectives on personalized therapy. Providing an explanation to support a microbiota-based diagnosis could guide decisions of clinical microbiologists*,* and has the potential to increase their confidence in the outcome of such decision support systems. This may facilitate the development of new diagnostic applications.

**Electronic supplementary material:**

The online version of this article (10.1186/s12859-017-1843-1) contains supplementary material, which is available to authorized users.

## Background

The human microbiota refers to the trillions of microorganisms living on and within our body, which are involved in many biological processes necessary to maintain human health. These microbial communities are associated with a wide range of disease states [1, 2], and hold a great promise for non-invasive diagnostics. The diagnostic potential of microbiota analysis has been extensively studied, for example, for disorders like inflammatory bowel disease (IBD) and diverticulitis [3–6] and has led to increasing efforts to develop clinical applications.

However, using microbiota for diagnostic purposes imposes several challenges. Studying the human microbiota is an active field of research. As knowledge is still accumulating, a full biological understanding of the underlying mechanisms is missing, limiting the diagnostic efforts to data-driven approaches, such as data mining. The data itself is high-dimensional and has high inter-personal variability. Consequently, identification of meaningful patterns is thwarted.

Therefore, machine learning (ML) tools are particularly useful in this field. Compared to humans, classifiers can more easily identify patterns in high-dimensional data, being able to learn to distinguish between categories (e.g., sick vs. healthy) based on examples. A trained classifier can then make predictions for new patients, accompanied by a probability reflecting its certainty. However, contrary to humans, classifiers usually do not provide an explanation to their propositions. In most cases they are considered as black boxes, taking features (e.g., microbial species) as input and returning a prediction. In a diagnostic setting, ML tools are able to assist us, but still cannot be blindly trusted.

Therefore, in order to effectively incorporate ML tools in microbiota-based diagnostics it is important to provide clinical microbiologists with a sensible, sample-specific *explanation* for every classifier prediction. This will allow them to weigh the outcome in the clinical and biological context, interpret it and even adjust specific therapy based on microbial species that appear to play a role in the diagnosis.

In this paper, we present how an idea originally proposed by Robnik-Šikonja and Kononenko [7] can be implemented for microbiota-based diagnostics. The method simulates input features as being unknown to measure their effect on the classification outcome. Consequently, each input feature is assigned a *relevance* value that reflects its importance for a specific decision of the classifier. With this approach, we aim to provide each patient a unique quantitative estimation of which species in their microbiota were used to determine their health status by the classifier. Given a trained classifier and a microbiota sample of a new patient, the species relevance ranking may help interpret why this patient was given a specific diagnosis, highlight species of interest, and guide treatment.

This method was applied on two microbiota datasets generated by IS-pro, a technique designed for microbiota profiling in clinical routine [8]. We further also describe how the calculated explanations can be validated and visualized.

## Methods

### Subjects and samples

Two balanced datasets were included: skin versus gut microbiota (SVG, *n* = 94) and gut microbiota of IBD patients versus healthy individuals (IBD, *n* = 112). Skin microbiota samples were collected by swabbing the inner lower arm of healthy adults. Swabs were stored in RTF buffer immediately after collection and later frozen at −20 °C. Gut microbiota was sampled from healthy children and children diagnosed with IBD according to the diagnostic Porto-criteria for pediatric IBD. Fresh fecal samples were collected in sterile containers and immediately stored in the freezer at −20 °C as was previously described [9].

### Microbiota profiling by IS-pro

All samples were analyzed by intergenic spacer (IS) profiling (IS-pro) [8, 9]. IS-pro is a PCR-based technique that differentiates bacterial species by the length of the 16S–23S rRNA in combination with phylum-specific fluorescently labeled PCR primers. Three phylum-specific primers were used: (1) *Bacteroidetes*, (2) *Firmicutes*, *Actinobacteria*, *Fusobacteria*, and *Verrucomicrobia* (FAFV), and (3) *Protebacteria*. For more details see the Additional file 1: Supplementary Methods.

### Preprocessing

Preprocessing was carried out with the IS-Pro proprietary software suite (IS-Diagnostics, Amsterdam, the Netherlands) and resulted in microbial profiles, presented as peak profiles. Each peak represents an IS fragment and is characterized by a color that corresponds to the phylum (or phyla). The length of the IS fragment, measured in nucleotides (nt), discriminates bacterial species, and its intensity, measured in relative fluorescence units (RFU), reflects the abundance. Intensity values were log2 transformed. Each peak was taken as a feature for classification. After excluding all-zero features, 914 and 1199 features were left for classification in the SVG and IBD datasets, respectively.

### Supervised classification

The following classifiers were used: Linear Support Vector Machine (SVM), Random Forest (RF), Nearest Shrunken Centroids (NSC) and Logistic Regression with L2 regularization (LR).

These classifiers are especially suitable for high-dimensional data as they apply regularization or dimensionality reduction. Examples of their applicability to human microbiota were previously described [10]. Implementation was done using packages of the scikit-learn 0.17.0 Python library: svm.SVC (with linear kernel and probabilistic outputs); neighbors.nearest_centroid.NearestCentroid (with a 0.1 shrink threshold and probabilities as computed in [11]); ensemble.RandomForestClassifier (with 50 trees), and linear_model.LogisticRegression (with L2 penalty).

Overall relevance measures of NSC were obtained per class by taking the difference between the global centroid and the class centroid, to which we compared our explanation for the predicted class. For the RF classifier, we used the importance ranking given by RandomForestClassifier.feature_importances. Note that using the weights of a linear SVM as an indication of feature importance is wrong and misleading [12]. Similarly, the coefficients of the LR classifier cannot be interpreted directly.

### Algorithm for explaining classifier decisions

Robnik-Šikonja and Kononenko [7] proposed that in order to measure how important a feature (e.g., a bacterial species) is, we can evaluate the classifier output when that feature is considered “unknown”. In our case, we seek to evaluate the decision the classifier would make if the information about a single bacterial species is not available. By simulating this for every feature individually, we obtain an overall ranking of the importance of each species for a sample-specific classification.

The authors proposed three strategies to evaluate the classifier while leaving one feature out: simply declare the feature as unknown (which only few classifiers allow), re-train the classifier without that feature (which is computationally unfeasible for clinical applications if there are many features), or approximately marginalize the feature out. We chose the latter for our analysis. Based on this strategy, we used the empirical distribution of the feature in the dataset, and replaced its value in the given sample with all other possible values it obtained in the dataset. The average prediction probability over those values was then used to determine the importance of the feature (see the Additional file 1: Supplementary Methods for the mathematical formulation). The algorithm returns a vector of the same size as the number of features, where each entry reflects the relevance of the respective input feature. A positive relevance value means that the feature value is a supportive evidence for the decision, and a negative relevance value expresses that the feature value constitutes evidence against the decision (and therefore evidence for the other class in a two-class problem like ours).

### Univariate vs. multivariate approach

The method described above is a *univariate* approach: to estimate a feature’s relevance, we marginalized out *only* this one feature. Intuitively, we would expect the classifier to stay robust to changes in only one feature, for example when features are redundant. In this case, it might be necessary to remove several features at once to have a noticeable effect on the prediction of the classifier. We propose a computationally feasible multivariate implementation for microbiota data in the Additional file 1: Supplementary Methods.

### Analytic validation of resulting explanations

When the data is poorly understood by humans, it is not straightforward to assess how good an explanation is. We propose a novel method for the analytic validation of the explanations, since we have not found any such method in literature. The relevance estimation method described above returns a relevance ranking which indicates a contribution measure of each feature. Features were sorted according to their relevance in ascending order (from features with negative influence on the class score, to irrelevant features, to the most important ones). Then, we successively marginalized out a growing number of features, starting with the least important ones, until no features were left. We used the predicted class for the analysis, and observed how the class probability changed based on the features' subset used for the classification. The class probability should rise when features with negative evidence for the predicted class are ignored, remain stable if features of zero importance are removed, and decline when highly discriminative features are ignored.

We also assessed our results based on pairwise correlations between relevance vectors of a sample calculated by different classifiers, as well as between sample-specific relevance measures and global ones.

## Results

In this study, we calculated explanations for individual classifier decisions using two microbiota datasets. Each sample is represented as a peak profile, composed of peaks representing different bacterial species and their abundances, which are taken as features for classification (see Methods).

The first dataset, denoted “SVG” (Skin Versus Gut), consisted of 47 skin microbiota samples that were classified against 47 gut microbiota samples (both of healthy subjects). Since the microbiota composition that inhabits skin or gut is very different, this classification task is relatively easy and can be directly interpreted by experts. This dataset was used to demonstrate how the method works and how the results can be presented, while allowing a qualitative evaluation of the method.

The second dataset, denoted “IBD”, consisted of 56 IBD gut microbiota samples (20 Ulcerative Colitis patients and 36 Crohn’s disease patients) classified against 56 healthy gut microbiota samples. This data is more complex and not so well understood, and classifiers outperform human expertise. It is those cases that are interesting for clinical applications, and for which explanations for classifier decisions are especially desirable.

The performance obtained by the classifiers in terms of prediction accuracy in a 10-fold cross-validation test was: SVM - 98% and 81%, RF - 99% and 81%, NSC - 99% and 79%, and LR - 100% and 78%, for SVG and IBD datasets, respectively.

Each classifier’s decision is accompanied by a probability value reflecting the certainty of the classifier (all of the above classifiers are either probabilistic or their output can be transformed to probabilities). To *explain* the decision, we assigned each input feature a *relevance value*. A positive value means that the feature’s value was supportive evidence *for* the predicted class, and a negative value means it was evidence *against* the predicted class.

### Skin vs. gut microbiota

This section presents the results for a simple case study: the SVG dataset.

#### Explaining a single prediction

**Fig. 1:**
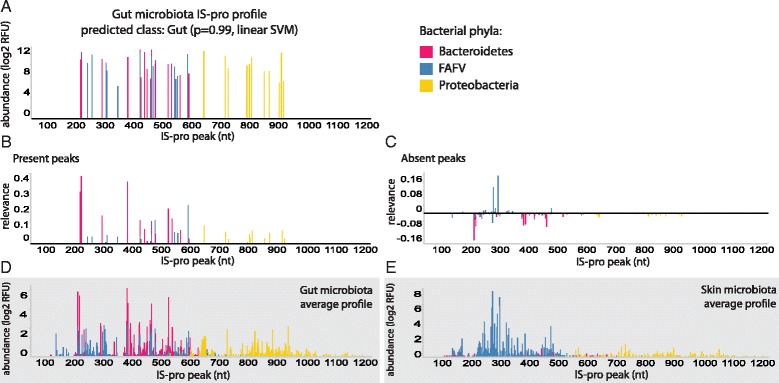
A gut microbiota sample and its peaks’ relevance values: (**a**) A gut microbiota sample: Peaks correspond to bacterial species; Peak heights correspond to abundance. (**b**) Relevance values calculated for each present peak in the profile in (**a**). (**c**) Relevance values calculated for each zero-valued peak in the profile in (**a**). (**d**) Average gut microbiota profile across all samples. (**e**) Average skin microbiota profile across all samples; FAFV: *Firmicutes*, *Actinobacteria*, *Fusobacteria* and *Verrucomicrobia*; Relevance was calculated based on SVM predictions

Figure 1 displays the output of the explanation algorithm for a *single* prediction of the classifier. For this example, we selected a gut microbiota sample correctly classified by a linear SVM. Bacterial species abundances (feature values) are shown in Fig. 1a, and the relevance values calculated for each feature are illustrated below (Fig. 1b and c). To facilitate interpretation, we split the relevance calculated for positive-valued features (present peaks, Fig. 1b) from that of zero-valued features (absent peaks, Fig. 1c), since zero-abundance peaks also hold information but are not visible in the profile and therefore can be visually confusing.

In general, for a single gut microbiota sample, we expect that common gut colonizers would obtain positive relevance values when they are found in the sample, and negative relevance values in case they are not detected. On the other hand, common skin colonizers should obtain negative relevance values when they are found in the sample and positive values otherwise. This was indeed observed for this example, with common members of the gut microbiota, such as *Bacteroides* and *Prevotella* species, being assigned positive relevance when they were found in the sample. This is indicated by the matching peaks between Fig. 1b and d. Prevalent skin species that were not detected in this sample, such as *Staphylococcus epidermidis*, also obtained positive relevance. This is indicated by the matching peaks between Fig. 1c and e. Table 1 lists the top ranked species according to absolute relevance, together with their presence status in this sample and the body habitat they usually colonize as a validation.

**Table 1:** Most relevant bacterial species for classification of the gut microbiota sample shown in Fig. 1

Species name	IS-pro peak(s)	Presence	Relevance	Common habitat
*Alistipes putredinis*	235	+	0.43	Gut
*Alistipes finegoldii*	396	+	0.4	Gut
	231	+	0.33	Gut
*Lachnospiraceae* sp.	605	+	0.25	Gut
*Bacteroides fragilis*	537	+	0.22	Gut
*Staphylococcus epidermidis*	313	–	0.2	Skin
*Odoribacter splanchnicus*	307	+	0.18	Gut
*Bacteroides* sp.	549	+	0.16	Gut
*Prevotella* sp.	438	+	0.15	Gut
*Lachnospiraceae* sp.	491	+	0.15	Gut
*Alistipes finegoldii*	230	–	−0.14	Gut
*Bacteroides vulgatus*	478	+	0.14	Gut
*Streptococcus mitis*	296	–	0.13	Skin
*Sutterella wadsworthensis*	661	+	0.12	Gut
unclassified *Bacteroidete*	455	+	0.09	Gut
unclassified *Proteobacterium*	932	+	0.09	–
*Escherichia coli*	735/828	+	0.08	Gut
unclassified *Firmicute*	558	+	0.07	Gut

’+’ and’-’ indicate whether a peak is present or absent in a sample, and needs to be coupled with the relevance value for interpretation. Ranking is based on absolute relevance values; Classification was done by a linear SVM

#### Meta-analysis of all samples

**Fig. 2:**
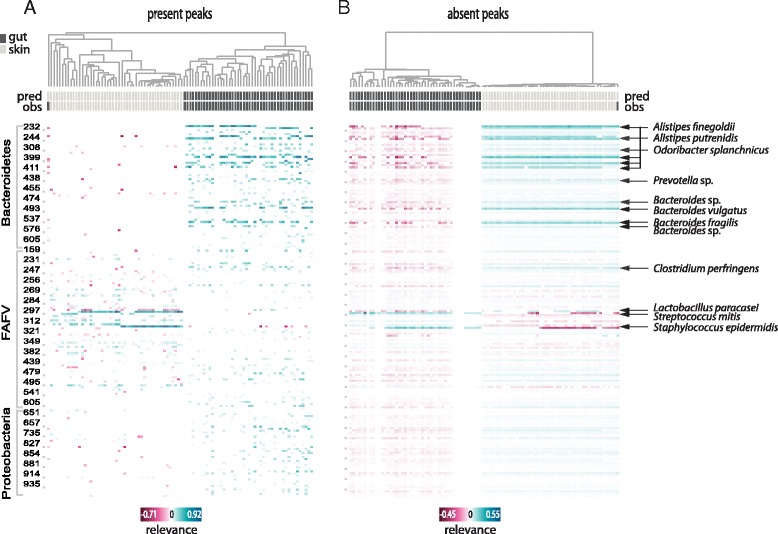
Skin microbiota vs. gut microbiota: Clustered heat maps of peaks’ relevance values across all samples. Columns correspond to samples, rows to peaks (or bacterial species). Top relevant species for classification are indicated. (**a**) Heat map including only the relevance calculated for positive-abundance peaks in each sample; (**b**) Heat map including only the relevance calculated for zero-abundance peaks in each sample. Only peaks with mean absolute relevance above 0.01 are shown in each heat map. Color key from pink to blue indicates relevance from low to high, respectively. For each sample the predicted class (pred) and the true class (obs) are indicated. Clustering is based on cosine correlation matrix followed by unweighted pair group method with arithmetic mean (UPGMA). Relevance values were calculated based on linear SVM predictions

While the proposed method is mainly aimed to explain single predictions, to validate it and gain insight into its behavior across the entire dataset, we calculated explanations for *every* sample in the dataset. These were summarized in heat maps (Fig. 2) to visualize the overall relevance of features in the dataset. To facilitate a visual interpretation, we again separated the present peaks (Fig. 2a) from the absent peaks (Fig. 2b). In both heat maps, the relevance values clustered the samples according to their (mostly correctly) predicted class. Prevalent colonizers of each habitat stood out with especially high or low relevance values, depending on the class and the presence status. Top ranked species, by means of average absolute relevance across all samples, are listed in Table 2.

**Table 2:** Most relevant bacterial species for classification of skin microbiota versus gut microbiota samples

Species name	IS-pro peak(s)	Common habitat	Ranked top 15 by:
*Alistipes finegoldii*	230/231/395/396/400/407	Gut	all
*Alistipes putredinis*	235/236	Gut	all
*Bacteroides vulgatus*	478/479	Gut	all
*Bacteroides fragilis*	537	Gut	all
*Clostridium perfringens*	235	Gut	all
*Streptococcus mitis*	296/297	Skin	all
*Staphylococcus epidermidis*	312/313	Skin	svm, nsc, lr
*Prevotella* sp.	437/438	Gut	svm, rf, lr
*Bacteroides* sp.	474	Gut	svm, rf, nsc
*Lactobacillus paracasei*	294	Gut	svm, nsc
*Staphylococcus epidermidis*	303	Skin	nsc, lr
*Streptococcus* sp.	321	Gut	nsc, lr
*Bifidobacterium adolescentis*	495	Gut	nsc, lr
*Clostridium* sp.	231	Gut	rf
*Odoribacter splanchnicus*	308	Gut	rf
*Streptococcus* sp.	318	Gut	rf
*Lactobacillus* sp.	478	Gut	rf
unclassified *Proteobacterium*	941	–	rf
*Lactococcus lactis*	344	Gut	nsc
*Propionibacterium acnes*	269	Skin	nsc

Ranking is based on absolute mean relevance values across all samples per classifier. Species are ordered according to the number of classifiers by which they were top-ranked. All - all classifiers; svm - linear SVM; rf - Random Forest; nsc - Nearest Shrunken Centroids; lr - L2-regularized Logistic Regression

### Healthy vs. IBD gut microbiota

While body habitats are relatively easy to classify, and the calculated explanations of the classification are suitable for a qualitative interpretation, real-life clinical dilemmas are usually much more complicated and much less straightforward to interpret. The following use-case, in which we classified gut microbiota samples of IBD patients against gut microbiota of healthy individuals, is a good example.

#### Explaining a single prediction

**Fig. 3:**
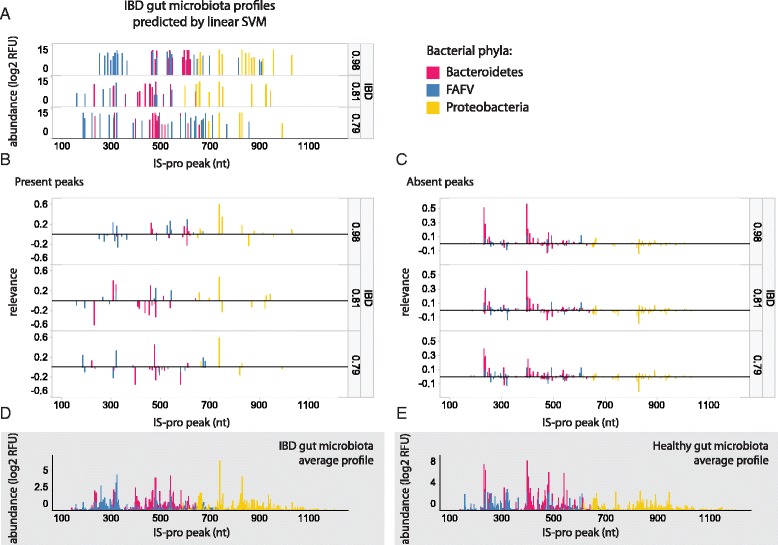
Three IBD gut microbiota samples and their corresponding peaks’ relevance values illustrate unique sample-specific relevance ranking. All samples were correctly classified, with the classifier’s probability values displayed per sample. (**a**) Gut microbiota samples: peaks correspond to bacterial species; Peak heights correspond to abundance. Classification probability is shown to the right of each profile. (**b**) Relevance values calculated for each present peak in the profiles in (**a**). (**c**) Relevance values calculated for each absent peak (i.e. zero-valued peak) in the profiles in (**a**). (**d**) Average IBD gut microbiota profile across all samples. (**e**) Average healthy microbiota profile across all samples FAFV: *Firmicutes*, *Actinobacteria*, *Fusobacteria* and *Verrucomicrobia*; Relevance was calculated based on SVM predictions

Figure 3 illustrates the relevance calculated for three selected IBD individual samples, all correctly classified by a linear SVM. Similar to Fig. 1, peak-specific relevance values are presented in reference to their abundance profile (Fig. 3a and b), which allows a straightforward interpretation of which peaks in the profile drove the classification towards the IBD class or against it. In a separate panel (Fig. 3c) the same is displayed for zero-abundance peaks. The most relevant species to explain the classifier’s decision for the top example are listed in Table 3.

**Table 3:** Most relevant bacterial species for the top IBD microbiota sample presented in Fig. 3

Species name	IS-pro peak(s)	Presence	Relevance
*Escherichia coli*	735	+	0.62
*Alistipes finegoldii*	396	–	0.57
*Alistipes finegoldii*	230	–	0.52
unclassified *Proteobacterium*	747	+	0.35
*Lachnospiraceae* sp.	606	+	0.3
*Alistipes putredinis*	235	–	0.29
unclassified *Firmicute*	326	+	−0.26
*Finegoldia magna*	306	+	0.24
*Parabacteroides distasonis*	461	+	0.23
*Morganella morganii*	855	+	−0.23
*Alistipes finegoldii*	400	–	0.21
*Bacteroides fragilis*	605	+	−0.21
*Escherichia coli*	827	+	0.19
*Alistipes putredinis*	236	–	0.18
*Streptococcus* sp.	321	+	0.17
unclassified *Firmicute*	525	+	0.17
*Bacteroides vulgatus*	479	–	0.16
*Bacteroides* sp.	594	+	0.16
*Alistipes finegoldii*	407	–	0.15
*Escherichia coli*	828	–	−0.14
*Bacteroides fragilis*	537	–	0.05

’+’ and’-’ indicate whether a peak is present or absent in a sample, and needs to be coupled with the relevance for interpretation. Ranking is based on absolute relevance values; Classification was done by a linear SVM

**Fig. 4:**
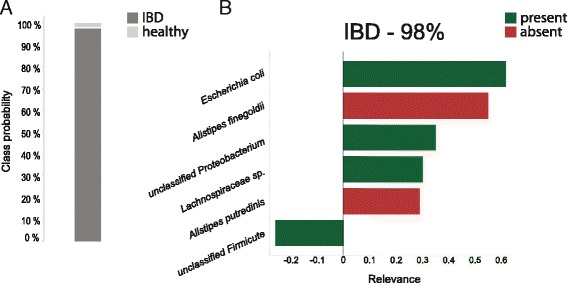
Output of a classifier without (**a**) and with (**b**) explanation

In a clinical context, such relevance ranking can improve the understanding of a classifier’s output by clinicians, and potentially support their decisions. Species that were assigned high relevance values may then guide an intervention according to their abundance. For example, in the top case presented in Fig. 3, determining a relevance cutoff of 0.25 would result in a short list of species that can be presented to clinical microbiologists, which is more informative than just presenting the decision (i.e. class probability) itself (Fig. 4). Based on their expertise, it may be advised that eliminating *Escherichia coli* and/or supplementing *Alistipes* spp. would be beneficial for this particular patient.

#### Meta-analysis of all samples

**Fig. 5:**
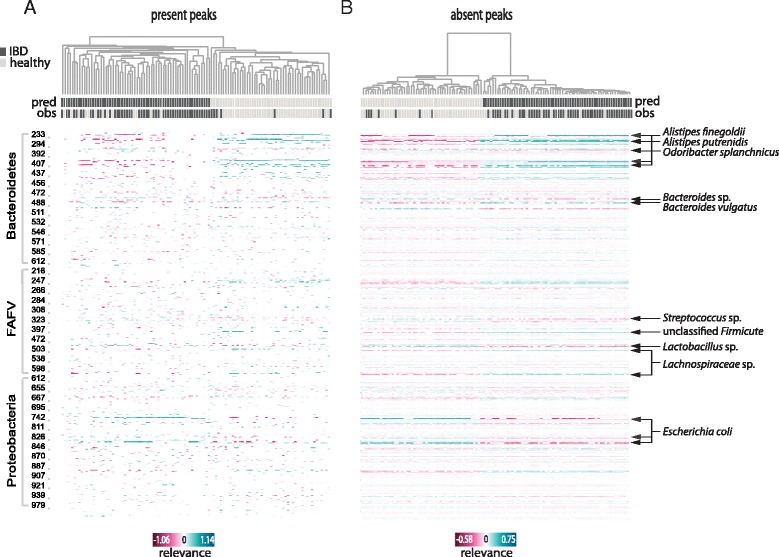
IBD gut microbiota vs. healthy gut microbiota: Clustered heat maps of peaks’ relevance values across all samples. Columns correspond to samples, rows to peaks (or bacterial species). Most relevant species for classification are indicated. (**a**) Heat map including only the relevance calculated for positive-abundance peaks in each sample; (**b**) Heat map including only the relevance calculated for zero-abundance peaks in each sample. Only peaks with mean absolute relevance above 0.01 are shown in each heat map. Color key from pink to blue indicates relevance from low to high, respectively. For each sample the predicted class (pred) and the true class (obs) are indicated. Clustering is based on cosine correlation matrix followed by unweighted pair group method with arithmetic mean (UPGMA). Relevance values were calculated based on linear SVM predictions

Figure 5 provides an overview of the relevance values calculated per peak for every sample in the dataset. The clustering of the samples matched the predicted classes, although it fitted less accurately the true class of the samples, since more samples were misclassified compared to the SVG dataset. The color pattern for prevalent species was complementary between the two classes and the presence (Fig. 5a) or absence (Fig. 5b) status, i.e., a prevalent peak that strongly supported one class also supported the other class by its absence, and vice versa. Top explanatory species by means of average absolute relevance for classification across all samples are shown in Table 4.

**Table 4:** Most relevant bacterial species for classification of IBD gut microbiota versus healthy gut microbiota samples

Species name	IS-pro peak(s)	Ranked top 15 by
*Alistipes finegoldii*	230/395/396/400/407	all
*Alistipes putredinis*	235/236	all
*Odoribacter splanchnicus*	307/308	all
unclassified *Firmicute*	396	all
*Escherichia coli*	735/821/827/828	all
unclassified *Bacteroidete*	251	svm, rf, nsc
*Lachnospiraceae* sp.	491/605	svm, rf, lr
*Bacteroides vulgatus*	479	svm, nsc
*Lactobacillus* sp.	478	svm, lr
*Streptococcus* sp.	318	svm, lr
*Bacteroides* sp.	474	svm, lr
*Streptococcus* sp.	321	nsc, lr
*Prevotella* sp.	438	rf, nsc
*Bacteroides fragilis*	537	rf, nsc
*Clostridium perfringens*	233/235	rf, nsc
*Lactobacillus* sp.	244	rf, nsc
unclassified *Firmicute*	156	rf

Ranking is based on absolute mean relevance values across all samples per classifier. Species are ordered according to the number of classifiers by which they were top-ranked. All - all classifiers; svm - linear SVM; rf - Random Forest; nsc - Nearest Shrunken Centroids; lr - L2-regularized Logistic Regression

### Discovering underlying subgroups within a class

**Fig. 6:**
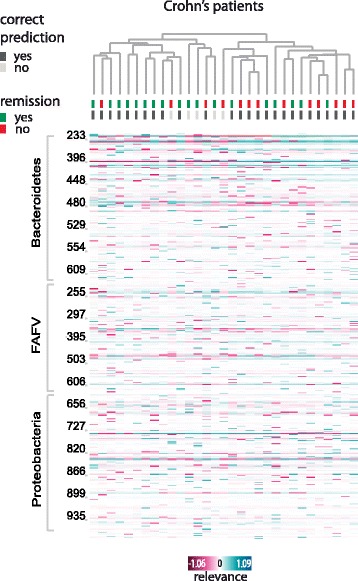
Response to exclusive enteral nutrition (EEN) in Crohn’s disease patients: a clustered heat map displaying peaks’ relevance values across Crohn’s disease patients who received EEN treatment. Columns correspond to samples, rows to peaks (or bacterial species). For each sample, it is indicated whether the prediction was correct (i.e. the sample was classified as ‘IBD’, dark grey) or not (the sample was classified as ‘healthy, light grey), and whether remission was induced in response to EEN (green) or not (red). The cluster on the left is enriched with patients with induced remission compared to the other cluster on the right (*p* = 0.04, chi-square test). Only peaks with mean absolute relevance above 0.01 are shown. Color key from pink to blue indicates relevance from low to high, respectively. Clustering is based on cosine correlation matrix followed by unweighted pair group method with arithmetic mean (UPGMA). Relevance values were calculated based on linear SVM predictions

IBD is a complex and multifactorial disease characterized by high between-patient variability in symptoms and response to treatment. This variability is attributed to various factors, including the intestinal microbiota composition. We show how the calculated explanations may be used to reveal underlying subgroups within a group of patients (Fig. 6). Most of Crohn’s disease patients in our cohort (32/36) were treated with exclusive enteral nutrition (EEN), and were evaluated again after a six-week period. These patients could be grouped into two clusters based on the calculated relevance values, with a significant enrichment of patients with induced remission in response to EEN in one cluster compared to the other cluster (*p* = 0.04, chi-square test).

### Analytic validation of explanations

Since the IBD dataset is poorly understood by humans, we sought for a way to analytically validate the results. Here, we propose a novel validation method that consists of subsequently removing a growing number of features, sorted by their relevance in ascending order. For a good explanation, the confidence of the classifier is expected to first go up (evidence against the prediction is removed), and then start to go down when important features are taken out. When all features are removed, the classifier has no information left and therefore its certainty should drop to 0.5 in a balanced dataset.

**Fig. 7:**
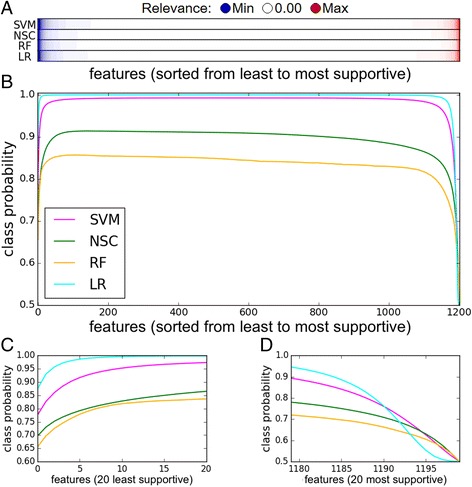
Validation of explanations: (**a**) Average feature relevance across all samples in the IBD dataset presented per classifier. Relevance values are sorted in ascending order, and color key from blue to red indicates relevance from low to high, respectively. (**b**) Validation plot: Features are sorted by relevance values and are plotted against the class probability, calculated when all features up to the current one at each point are ignored. The curve of each classifier is averaged over all samples. (**c**) and (**d**) The same curve as in (**b**), zoomed in on the first 20 (i.e., least support for predicted class) and last 20 (i.e., most support for the predicted class) features, respectively

The average validation results for the IBD dataset are presented in Fig. 7. Only a small number of features was very relevant (either as positive or negative evidence for the predicted class, Fig. 7a). Most features had a near-zero relevance score, and were therefore not important for the decision. We observed a rise in certainty when features with high negative relevance were removed, followed by a strong decline in response to removal of features with top-ranked positive evidence (Fig. 7b). In the middle range, where features with near-zero relevance were excluded, the validation curve stagnates, indicating that removing them had no influence on the classification.

In addition, we validated our results by comparing explanations calculated by different classifiers by sample, and comparing sample-specific explanations of a classifier with the respective model’s global ranking (if available by the classifier). The correlation was higher when calculated within-classifier, i.e. between the sample-specific relevance vectors and the global importance ranking of the same classifier, than between sample-specific relevance vectors of different classifiers (Additional file 2: Figure S1).

## Discussion

In this study, we present how a method aimed to explain individual classifier decisions can be applied to human microbiota data to facilitate the interpretation of microbiota-based predictions in a biologically meaningful context. Our algorithm is based on a method originally proposed by Robnik-Šikonja and Kononenko [7]. It generates a sample-specific feature relevance ranking that, combined with an abundance profile, allows us to reason about the importance of different species in driving a prediction. This gives an insight into the specific pathogenic perturbations of the microbiota of individual patients, which in turn may allow adjusting personalized treatment, supplementing or targeting specific species.

We first tested our approach on an easily classifiable dataset consisting of gut and skin microbiota samples. Species ranked highly relevant were identified as common inhabitants of each of these body sites [13]. Common gut colonizers, such as *Bacteroidetes* spp. and *E. coli*, were supportive evidence for the ‘gut’ class. Skin samples presented an opposite image, with dominant skin bacteria, such as *S. epidermidis* and *Streptococcus mitis*, driving the classification by their presence. It is important to note that evidence for or against a class can be found in the presence of certain species, but also in their absence.

Assessing the explanations for the ‘IBD versus healthy’ case was more challenging. Although findings of aberrations in the microbiota composition of IBD patients are well established [14–16], they are not consistent regarding specific species. Increased amounts of mucosa-associated *E. coli*, which was assigned high relevance values by all classifiers tested, were indeed previously detected in IBD patients [17]. We also compared our results to a recent study^1^ [9] in which a core microbiota, prevalent and stable over time, was identified in healthy children. Interestingly, strong evidence for the IBD class was given by the absence of peaks originating from species described as members of the pediatric healthy core microbiota, such as certain *Alistipes*, *Bacteroides* and *Prevotella* species. These species were mostly absent from IBD samples, and were assigned consistently high relevance values, which resulted in high dendrogram distances between the two clusters that appear in the heat map displaying the absent peaks (Fig. 5b). In general, in all heat maps, clustering based on the calculated relevance values separated the samples mostly according to their predicted class, which means the relevance values explain the classifier decisions well.

To analytically validate this, we proposed a method that estimates how the classifier’s probability for a class changes when features of varying relevance are excluded. This approach brings value since it is not always straightforward to assess how good an explanation is, especially when we lack knowledge about the domain. A good explanation means that the certainty of a classifier would decline only when high relevance features are ignored. Indeed, this was observed for all the classifiers we tested in the IBD dataset. We could rank features correctly according to their relevance even when a classifier was less certain about its decisions and resulted in lower probabilities, like in the case of RF (Fig. 7). In our case, the set of important features for one individual sample was quite small, meaning that a short list of the most important species could be effectively presented to a physician. Note that although the set of important features was rather small for an *individual* classification, this does not mean that the classifier relies only on those, and that they hold all the information. The set of important features varied widely between samples (Figs. 2 and 4), which corroborates the need for a *sample-specific* explanation method for microbiota data.

Still, sample-specific explanations were highly correlated with the global feature rankings at the model level (Additional file 2: Figure S1), indicating that our method conforms to the general rankings. Some discrepancies occur when comparing explanations between the different classifiers, which represent different algorithm concepts; SVM and LR are linear classifiers, RF is an ensemble of decision trees, and NSC relies on the distance between data points to class centroids. While there may be several solutions to a problem and different classifiers can make different mistakes, we can assume that the better the prediction accuracy of a classifier is, the closer the explanation of its decision is to the true difference between the classes. Nonetheless, in practice it is also important to pay attention to overfitting, which is a common concern in small microbiota datasets. While in our experiments we did not observe overfitting, it is important to take into account that in such cases the classifier may fit to random noise in the data, and the explanations might not be sensible.

The calculated relevance can also be used to reveal underlying subgroups within a patient group. As an example, we focused on response to EEN, an effective therapeutic measure to induce remission in active Crohn’s disease pediatric patients. EEN consists of a complete liquid diet [18] and leads to the induction of remission in approximately 85% of patients. In our cohort, remission was induced in 18 out of 32 patients. While several factors may influence the response to EEN, including disease duration and location, a biomarker to predict the response or to adjust an individualized application of EEN in children is not yet available. Our results suggest a role of the microbiota in the response to EEN, and illustrate the potential the explanations hold to unveil patterns in the data.

Several other techniques also aim to interpret the learning of a classifier. These are mostly focused on feature ranking and feature selection, which are global methods, independent of a specific input sample. An *individual* explanation may contribute to specific interpretation that can lead to customized clinical decisions, which are especially desired in microbiota analysis due to its highly personalized composition. However, the evaluation of the contribution in practice of such a method to support clinical decision making has not yet been conducted, which is a limitation of this study. Such evaluation should be carried out as part of future work, similarly to the work done by Lou et al. [19].

A common method for instance-specific relevance estimation is the sensitivity analysis [20], where the relevance of each feature of a particular input sample is given by the partial derivative of the output with respect to this feature. If a classifier’s decision is sensitive to the input (i.e., small changes in the feature lead to a large change in output probability), a high relevance value is assigned to that feature. This method requires the estimation of partial derivatives of the output with respect to the input, which for some classifiers may require (more expensive) empirical analysis. Moreover, in some cases, like in the case of linear SVM, the sensitivity analysis is not applicable, since it returns the weight vector, which is an incorrect explanation of feature importance. Furthermore, it cannot give insight into which features constitute evidence for or against a class, but only how important they are in an absolute sense. Yet, if the partial derivatives can be estimated analytically, it is faster and easier to implement than the method we used.

We hypothesized that since the microbiota is an ecological system, in which different species continuously interact, multiple species should be considered by the algorithm at once. In a follow-up paper [21], Štrumbelj and Kononenko suggested to marginalize out all possible subsets of features. However, neither this approach nor its approximation [22] are feasible for high-dimensional data. To circumvent the computational complexity, we implemented a multivariate method based on a sensible selection of subsets from the power-set of all features. However, the explanations were not affected by this extension, and we therefore concluded that the univariate approach is valid in our case. Nonetheless, more sophisticated classifiers (such as neural networks) might be more sensitive to feature correlations, and in such cases the multivariate approach would become valuable.

## Conclusions

The human microbiota consists of promising biomarkers for various disease states, but to facilitate the development of new diagnostic applications, results need to be reported in an intelligible way. We showed that an added explanation to each prediction allows a more extensive analysis, which in turn may allow clinical microbiologists to make informed decisions. The benefit of an explanation alongside a classifier’s decision is manifold: it may assist in treatment guidance for individual patients and can help improve clinical expertise, with new patient subgroups discovered. Finally, it holds potential to increase the understanding of classifiers’ outputs, which is key for their incorporation in clinical practice.

## Additional files

Additional file 1: Supplementary methods. (DOCX 20 kb)

Additional file 2: Figure S1. Correlation calculated between different relevance measures. Spearman’s rank correlation was calculated between sample-specific explanations calculated by different classifiers, and between sample-specific relevance measures and global ones at the model level, when available by the classifier (RF and NSC). Top: SVG dataset, bottom: IBD dataset. Left: Between-classifier correlations. Correlations are shown as absolute values, since two classifiers with oppositional predictions would have negatively correlated relevance measures. Right: Within-classifier correlations. For each sample, the correlation was calculated between the sample-specific explanation and the global relevance measures as given by the classification algorithm (for NSC and RF). The correlations between classifiers are higher in the SVG dataset than in the IBD dataset, probably because the classifiers make more mistakes on a harder classification task (IBD vs. healthy), which leads to wrong explanations (also for correct predictions). Since different classifiers make different mistakes, the overall correlation is lower. In both datasets, the highest correlation is obtained between the SVM and LR classifiers. The highest discrepancy occurred between RF and the rest of the classifiers, which could be attributed to the conceptual differences between the algorithms. Sample-specific rankings were highly correlated with the global rankings of the NSC and RF classifiers, indicating that the explanations are coherent with the model-level rankings. Differences still occur as sample-specific explanations are tailored to a single sample, and each microbial fingerprint gets a unique relevance ranking, as is shown by the three examples displayed in Fig. 3. (TIFF 415 kb)

## Abbreviations

EEN: Exclusive enteral nutrition
IBD: Inflammatory bowel disease
LR: Logistic regression
ML: Machine learning
NSC: Nearest shrunken centroids
RF: Random forest
RTF: Reduced Transport Fluid.
SVG: Skin versus gut
SVM: Support vector machine

## Footnotes

1 of which our healthy cohort is a subset
