# Supplementary material for: Interpretation of microbiota-based diagnostics by explaining individual classifier decisions
Source: BMC Bioinformatics. 2017 Oct 4;18:441. doi: 10.1186/s12859-017-1843-1 (PMC5628491; doi:10.1186/s12859-017-1843-1)
Supplement: Supplementary file 1 — Supplementary methods. (DOCX 20 kb) [file 12859_2017_1843_MOESM1_ESM.docx]

**Text S1: Supplementary Methods**

### *Microbiota Profiling by IS-pro*

Microbiota profiling was performed by IS-pro. First, 300μl lysis buffer was added to an Eppendorf container containing 0.5 gr fecal sample. This mixture was vortexed and shaken for 5 min at room temperature, and then centrifuged for 2 min at 13,000 RPM. The supernatant was then transferred to a DNA isolation vial, and magnetic silica beads were added according to the routine protocol. Bacterial DNA was isolated by an automated isolation procedure (EasyMag, Biomerieux, Marcy l’Etoile, France) according to the manufacturer’s instructions. Amplification of IS regions was performed with the IS-pro assay (IS-Diagnostics, Amsterdam, the Netherlands) according to the protocol provided by the manufacturer. IS-pro differentiates bacterial species by the length of the 16S-23S rRNA with phylum-specific fluorescently labeled PCR primers. In short, DNA was eluted in 110μl buffer and stored at 4ºC until PCR amplification. Then, diluted DNA was amplified in two standardized multiplex PCR amplifications: first includes phylum-specific primer sets for the phyla: (1) *Firmicutes*, *Actinobacteria*, *Fusobacteria*, and *Verrucomicrobia* (FAFV) and (2) *Bacteroidetes*; the second includes phylum-specific primer sets for *Proteobacteria*. Subsequently, 5μl of PCR product was mixed with 20μl formamide and 0.2μl Mapmaker 1500 ROX labeled size marker (custom made by BioVentures, Murfreesboro, TN, USA). PCR products were then separated based on their lengths in an ABI Prism 3130XL Genetic Fragment Analyzer (Applied Biosystems Carlsbad, California, USA).

*Algorithm for Explaining Classifier Decisions*

A Univariate Approach

To measure how important a feature $x_{i}$ is ($1\leq i\leq D$, where $D$ is the number of features), we can evaluate the classifier output $p(c|\boldsymbol{x})$ when $x_{i}$ is unknown, i.e., estimate$p(c|\boldsymbol{x}\backslash_{i})$ where $c$ is the class of interest. Robnik-Šikonja and Kononenko [7] proposed to approximately marginalize the feature out - in

$p(c|\boldsymbol{x}\backslash_{i}) = \sum_{x_{i}} p(x_{i}|\boldsymbol{x}\backslash_{i})p(c|\boldsymbol{x}\backslash_{i},x_{i})$ (1)

they suggested to approximate $p(x_{i}|\boldsymbol{x})\approx p(x_{i})$. This makes the evaluation feasible, since modeling $p(x_{i}|\boldsymbol{x}\backslash_{i})$ can be difficult, and for microbiota data no appropriate probabilistic model is available. For the marginal distribution $p(x_{i})$ we used the empirical distribution, so that

$p(c|\boldsymbol{x}\backslash_{i})=\frac{1}{N}\sum_{x_{i}\in X_{i}^{train}} p(c|\boldsymbol{x}\backslash_{i},x_{i})$ (2)

where *N* is the number of training instances and $X_{i}^{train}$ is the set of all values feature $x_{i}$ takes in the training set. After estimating$p(c|\boldsymbol{x}\backslash_{i})$, this value can be compared to the original prediction,$p(c|\boldsymbol{x})$. We used the authors’ suggestion of evaluating the *weight of evidence*,

$\text{WE}_{i}\left( c | \boldsymbol{x} \right)=\log_{2} \text{odds}\left( c | \boldsymbol{x} \right)-\log_{2} \text{odds}\left( c | \boldsymbol{x}\backslash_{i} \right)$ (3)

where $\text{odds}(z)=\frac{p(z)}{1-p(z)}$ .

The algorithm returns a $D$-dimensional vector **WE**, reflecting the relevance of each feature$x_{i} (1\leq i\leq D)$. (Note: to avoid problems with zero-valued probabilities, the Laplace correction $p\leftarrow\frac{pN+1}{N+K}$ was used, where $K$ is the number of classes.)

A Multivariate Approach

A full multivariate approach (marginalizing out all possible feature subsets) is computationally unfeasible, we therefore propose here a method in which only a group of (wisely chosen) feature sets is used. In contrast to the univariate approach, the relevance of a feature is now defined given the weight of evidence not of single features but of feature subsets $S=\{S_{1},...,S_{n}\}\subseteq P(\boldsymbol{x})$ from the power set $P\left( \boldsymbol{x} \right)$ of all features, where each $S_{k} (1\leq k\leq n)$ is a set of features $\boldsymbol{x}'\subseteq\boldsymbol{x}$.

Such an approach requires first to express the relevance of a *single* feature, by combining results obtained from marginalizing out different overlapping feature subsets. We therefore define the relevance of each feature $x_{i}$ as follows:

$\text{rel}_{x_{i}}=\frac{1}{M}\sum_{x_{i}\in S_{k},S_{k}\in S} \log_{2} \text{odds}(c|(\boldsymbol{x}\backslash S_{k})\cup x_{i})-\log_{2}\text{odds}(c|\boldsymbol{x}\backslash S_{k})$(4)

where $M$ is the number of sets $S_{k}$ that hold $x_{i}$. By this, we evaluate the impact of $x_{i}$ also in combination with other features. Redundant features would now result in higher relevance estimation compared to the univariate approach.

To reduce the computational complexity, a limited number of subsets was wisely selected, based on *a priori* knowledge. The datasets used in this study consisted of peaks that co-occur because they belong to the same bacterial species or to a subset of species that are typically present in samples that belong to the same class based on literature [9,13]. This information, combined with peak correlation and co-occurrence counts, was used to form sensible subsets of features. By incorporating technical and biological properties of our data, we tested the following subsets: random subsets of different size, windows of size 3-8 (to correct for machine errors), sets of peaks that belong to the same species, known core species that characterize each class, correlated pairs of features, and co-occurring pairs of features.
