# Supplementary figures and images for: Interpretation of microbiota-based diagnostics by explaining individual classifier decisions
Source: BMC Bioinformatics. 2017 Oct 4;18:441. doi: 10.1186/s12859-017-1843-1 (PMC5628491; doi:10.1186/s12859-017-1843-1)

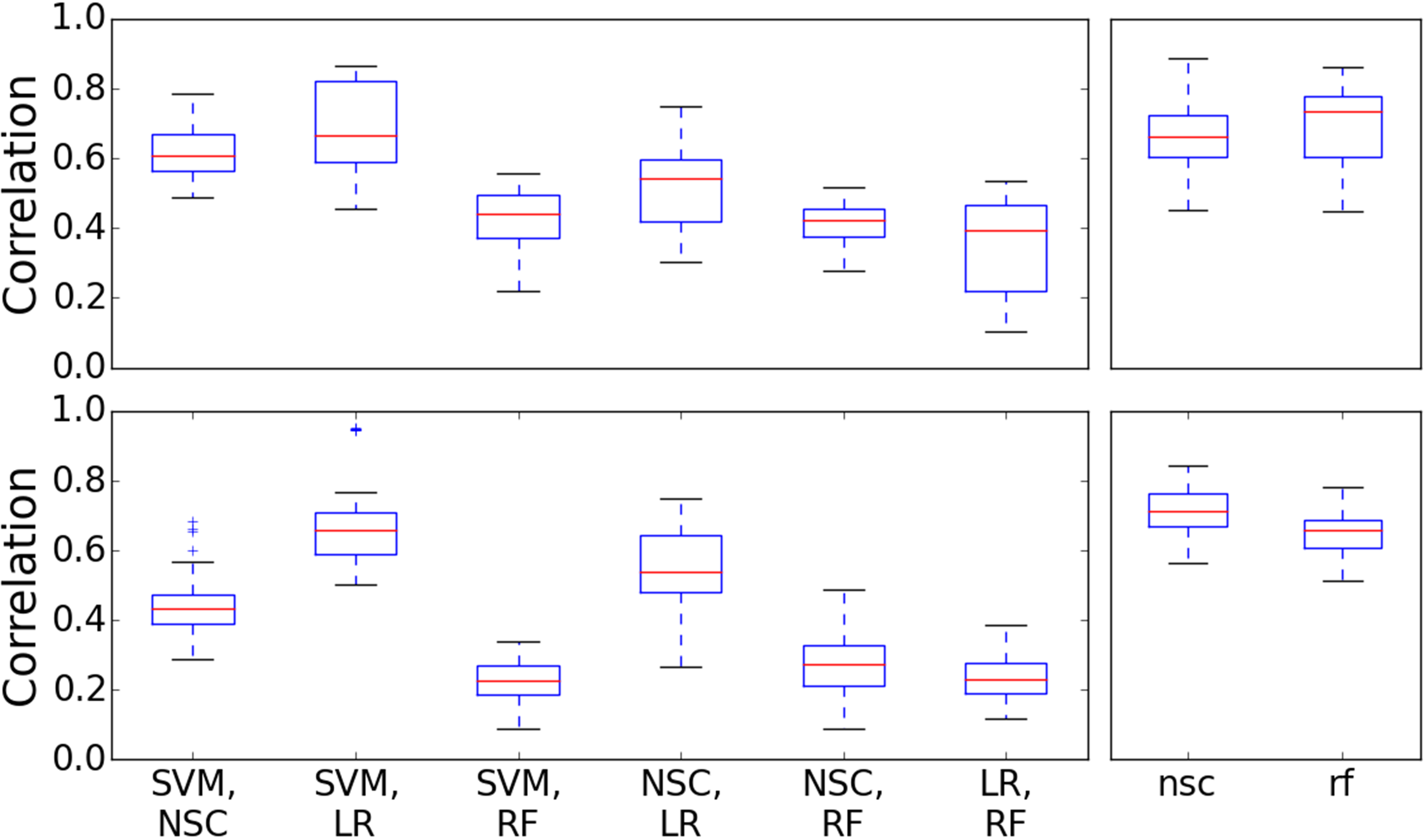

Supplement: Supplementary file 2 — Correlation calculated between different relevance measures. Spearman’s rank correlation was calculated between sample-specific explanations calculated by different classifiers, and between sample-specific relevance measures and global ones at the model level, when available by the classifier (RF and NSC). Top: SVG dataset, bottom: IBD dataset. Left: Between-classifier correlations. Correlations are shown as absolute values, since two classifiers with oppositional predictions would have negatively correlated relevance measures. Right: Within-classifier correlations. For each sample, the correlation was calculated between the sample-specific explanation and the global relevance measures as given by the classification algorithm (for NSC and RF). The correlations between classifiers are higher in the SVG dataset than in the IBD dataset, probably because the classifiers make more mistakes on a harder classification task (IBD vs. healthy), which leads to wrong explanations (also for correct predictions). Since different classifiers make different mistakes, the overall correlation is lower. In both datasets, the highest correlation is obtained between the SVM and LR classifiers. The highest discrepancy occurred between RF and the rest of the classifiers, which could be attributed to the conceptual differences between the algorithms. Sample-specific rankings were highly correlated with the global rankings of the NSC and RF classifiers, indicating that the explanations are coherent with the model-level rankings. Differences still occur as sample-specific explanations are tailored to a single sample, and each microbial fingerprint gets a unique relevance ranking, as is shown by the three examples displayed in Fig. 3. (TIFF 415 kb) [file 12859_2017_1843_MOESM2_ESM.tif]
